# Visuomotor expertise in volleyball: saccadic latency and absolute alpha power

**DOI:** 10.3389/fpsyg.2026.1788088

**Published:** 2026-06-30

**Authors:** Renan Vicente, Juliana Bittencourt, Renato Fonseca, Giovanna Zanchetta, Alexandre Vasconcelos, Carlos Amoroso, Marcos Machado, Elida Costa, Isabelle Fernandes, Victor Marinho, Jesse Di Giacomo, Eduardo Nicoliche, Danielle Aprigio, Marcelo Nobre, Marco Orsini, Victor Hugo Bastos, Silmar Teixeira, Antonio E. Nardi, Mauricio Cagy, Bruna Velasques, Pedro Ribeiro, Henning Budde

**Affiliations:** 1 Brain Mapping and Sensory Motor Integration, Institute of Psychiatry, Federal University of Rio de Janeiro, Rio de Janeiro, Brazil; 2 Postgraduate Program in Psychiatry and Mental Health (PROPSAM), Federal University of Rio de Janeiro, Rio de Janeiro, Brazil; 3 Veiga de Almeida University, Rio de Janeiro, Brazil; 4 School of Physical Education and Sports, Federal University of Rio de Janeiro, Rio de Janeiro, Brazil; 5 Laboratory of Neurophysiology and Neuropsychology of Attention, Institute of Psychiatry, Federal University of Rio de Janeiro, Rio de Janeiro, Brazil; 6 Brain Mapping and Plasticity Laboratory, Federal University of Piauí (UFPI), Parnaíba, Brazil; 7 Biomedical Engineering Program, COPPE, Federal University of Rio de Janeiro, Rio de Janeiro, Brazil; 8 Bioscience Department, School of Physical Education, Federal University of Rio de Janeiro, Rio de Janeiro, Brazil; 9 Institute for Systems Medicine (ISM), MSH Medical School Hamburg University, Hamburg, Germany

**Keywords:** absolute alpha power, cognition, EEG, neural efficiency, reaction time, saccadic latency, volleyball

## Abstract

This study investigated differences in saccadic latency and absolute alpha power during eye movement preparation between young volleyball athletes and non-athletes. Two experimental paradigms were employed: fixed (memory-guided) and random (stimulus-guided) saccadic tasks. Thirty participants (15 athletes, 15 non-athletes) performed these tasks while electroencephalographic (EEG) activity was recorded from prefrontal and frontal cortical regions. Behaviorally, saccadic latency was significantly shorter in the random task compared to the fixed task; however, no significant group differences were observed. Electrophysiological analyses demonstrated that volleyball athletes consistently exhibited lower absolute alpha power at frontal and prefrontal electrode sites (Fp2, F7, F8, Fz, F4), suggesting heightened cortical engagement during visuomotor preparation. Both groups showed reduced alpha power in the random condition at lateral frontal sites (F7, F8), indicating enhanced attentional allocation to unpredictable stimuli. These findings imply that sports expertise may manifest as subtle neural adaptations not easily detected by behavioral measures like reaction time, thus underscoring the value of EEG in elucidating training-induced neurophysiological changes. Future research should utilize ecologically valid tasks and higher-density EEG recordings to further delineate the neural mechanisms underpinning sports expertise.

## Highlights

•Behavioral performance did not differ between groups, suggesting that electrophysiological measures, such as EEG, may offer greater sensitivity than reaction time in detecting training-related neural adaptations.

•Volleyball athletes exhibited reduced alpha power in frontal and prefrontal regions, indicative of enhanced cortical engagement during saccadic preparation. •Task effects were observed exclusively at lateral frontal sites, with lower alpha power in the random condition compared to the fixed condition, reflecting the increased attentional demands associated with unpredictability. •Volleyball training was associated with distinct patterns of frontal activation, revealing neural adaptations that were not captured by behavioral measures.

## 1 Introduction

Saccadic eye movements are rapid, ballistic gaze shifts between fixation points, forming a core oculomotor mechanism for visual attention and information acquisition (Yarbus, 1967). Their behavior is modulated by cognitive processes such as working memory and attentional control (Land, 2009; Schütt et al., 2019; Wollenberg et al., 2020). Presaccadic attention enhances processing at the saccade target before movement onset, optimizing temporal perception and visual efficiency (Coop et al., 2024; Yan et al., 2025).

Saccadic latency, defined as the interval between stimulus presentation and saccade onset, is a reliable marker of visuomotor efficiency and central response speed (Leigh and Zee, 2015). It reflects attentional engagement, motor planning, and sensorimotor integration (McDonough and Siegel, 2018; Topfstedt et al., 2023).

Quantitative electroencephalography (qEEG), with high temporal resolution, is widely used to investigate neural correlates of sports performance (Bazanova and Vernon, 2014; Thompson et al., 2008). Alpha-band (8–13 Hz) spectral analysis reveals mechanisms of visual control, motor preparation, and cognitive efficiency (Del Percio et al., 2019; Klimesch, 1999). Absolute alpha power indexes cortical activation or inhibition, decreasing in task-relevant regions and increasing in those suppressed (Lu et al., 2020; Pfurtscheller et al., 1996).

Saccadic efficiency is crucial for rapid decision-making in sports (Jafarzadehpur et al., 2007). In volleyball, athletes must sustain attention to dynamic visual cues (Zhang et al., 2024), executing fast, precise saccades that support anticipation, decision-making, and motor control (Buscemi et al., 2024; Leung et al., 2021). Athletes in perceptually demanding sports often outperform non-athletes in sensory-motor tasks (Bisagno et al., 2022; De Waelle et al., 2021), reflecting enhanced executive functions, visuomotor integration, and training-induced neuroplasticity (Krenn et al., 2018; Neubauer and Fink, 2009). Recent studies also report faster, more accurate saccades in athletes, indicating superior visual processing efficiency (Chen et al., 2024; Tatara et al., 2024; Vicente et al., 2023).

Empirical evidence shows distinct alpha activity patterns between athletes and non-athletes. In experienced athletes, alpha modulation supports the neural efficiency hypothesis, suggesting optimal performance with reduced energetic expenditure (Babiloni et al., 2010; Neubauer and Fink, 2009). Simple tasks elicit lower alpha power, whereas complex tasks often increase frontal or parietal alpha, reflecting task-specific strategies linked to expertise (Babiloni et al., 2008; Wolf et al., 2014; Wu et al., 2013). Alpha activity in motor cortices indicates reduced inhibition and heightened readiness, and neurofeedback training has been associated with improved athletic performance (van Boxtel et al., 2024; Wilken et al., 2025). Elite athletes also display attenuated alpha reactivity and EEG markers of enhanced neural efficiency (Del Percio et al., 2011; Mikicin et al., 2015).

Assessing saccadic latency alongside absolute alpha power offers a comprehensive measure of visuomotor efficiency and neural preparation in volleyball athletes vs. non-athletes. Memory-guided and stimulus-guided saccade paradigms enable evaluation of how cognitive demands affect behavioral and electrophysiological outcomes (Bittencourt et al., 2013; Brown et al., 2004). This integrative approach is supported by evidence linking attention, eye movements, and neural activity, including presaccadic effects on temporal perception (Yan et al., 2025) and neural enhancement (Coop et al., 2024).

This study compared volleyball players and non-athletes in saccadic latency and absolute alpha power during eye movement preparation under fixed (memory-guided) and random (stimulus-guided) conditions. The aims were to assess group differences in latency and examine frontal and prefrontal alpha power. We hypothesized that athletes would exhibit shorter latencies and lower alpha power (8–13 Hz), indicating enhanced visuomotor efficiency, attentional focus, and saccadic preparation.

## 2 Material and methods

### 2.1 Participants

Thirty volunteers participated and were divided into two groups: 15 volleyball athletes (seven males, eight females; mean age = 15.8 ± 0.2 years) and 15 non-athletes (seven males, eight females; mean age = 16.2 ± 0.3 years). An independent samples *t*-test showed no significant age difference between groups (*p* > 0.05). All participants were right-handed, as assessed by the Edinburgh Handedness Inventory (Oldfield, 1971), and had normal or corrected-to-normal vision. Exclusion criteria included any history of psychiatric or neurological disorders and the use of psychoactive or psychotropic substances.

Sample size estimation was based on previous studies using similar experimental designs (Del Percio et al., 2007a; Di Russo et al., 2005a), with a deliberate increase in participant numbers compared to prior work. Athletes were recruited from a professional volleyball club and had an average of 5.0 ± 2.8 years of training experience. The control group consisted of students from local schools who did not engage in regular sports practice. All participants abstained from substances known to affect brain activity (e.g., tobacco, coffee, alcohol, caffeine-containing foods, and medications) for at least 14 h before and during testing. Ophthalmological examinations confirmed normal vision, and medical screening excluded neurological, motor, or other conditions contraindicating participation. The study was approved

by the local ethics committee, and written informed consent and assent were obtained from all participants and their legal guardians, in accordance with the Declaration of World Medical Association (1964).

### 2.2 Experimental procedure and task

**Figure 1:**
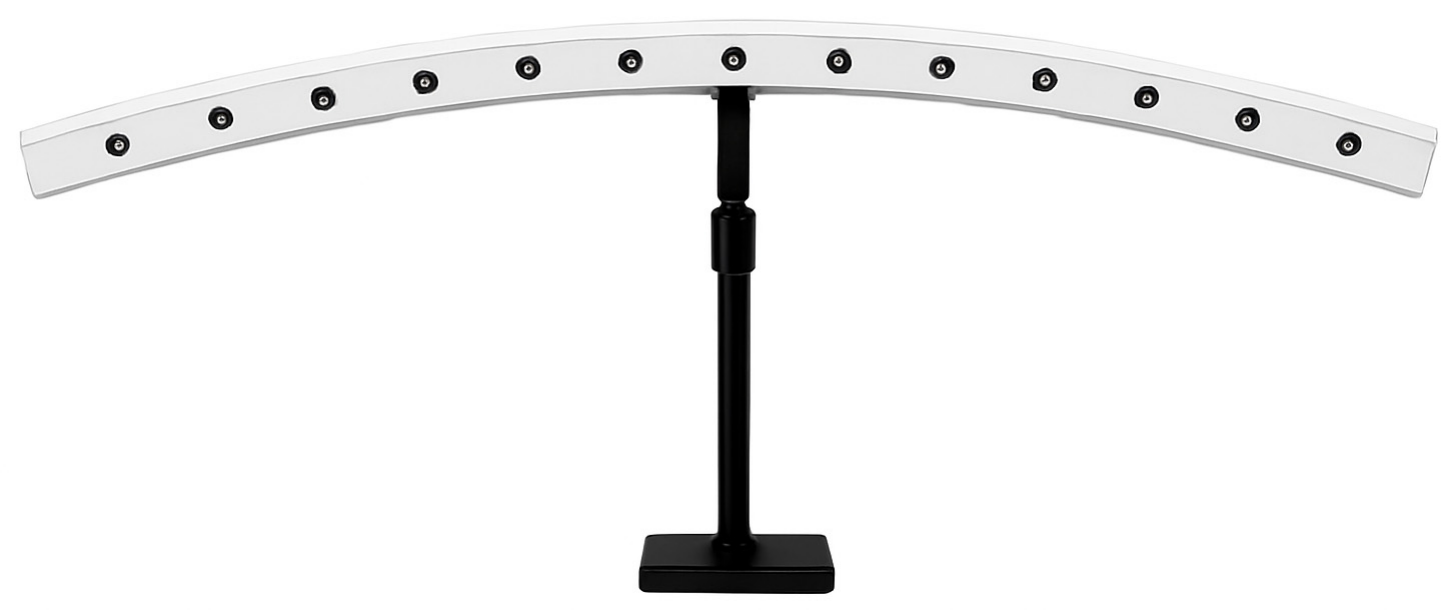
Model of the light-emitting diodes (LEDs) bar setup.

Participants were seated in a room with controlled lighting, acoustic isolation, and electrical grounding. A 120 cm bar containing 13 light-emitting diodes (LEDs) was positioned 100 cm in front of the participants, see Figure 1. The central LED served as the fixation point, flanked by six LEDs on each side. The six LEDs on each side were spaced approximately 9.62 cm apart, subtending the following angular eccentricities from the central fixation point: LED 1 = 5.5°, LED 2 = 10.9°, LED 3 = 16.1°, LED 4 = 21.1°, LED 5 = 25.7°, and LED 6 = 30.0°. Stimulus presentation and reaction time recording were controlled by SEM Acquisition software. Saccadic latency was measured concurrently with electrooculogram (EOG) recordings, using two 9 mm electrodes placed bipolarly at the outer canthi to capture horizontal eye movements (hEOG). The EOG signal was used exclusively for saccadic onset detection and not for saccade amplitude estimation. Saccadic onset was identified by the peak of the second derivative of the EOG signal, above a 75th-percentile threshold.

**Figure 2:**
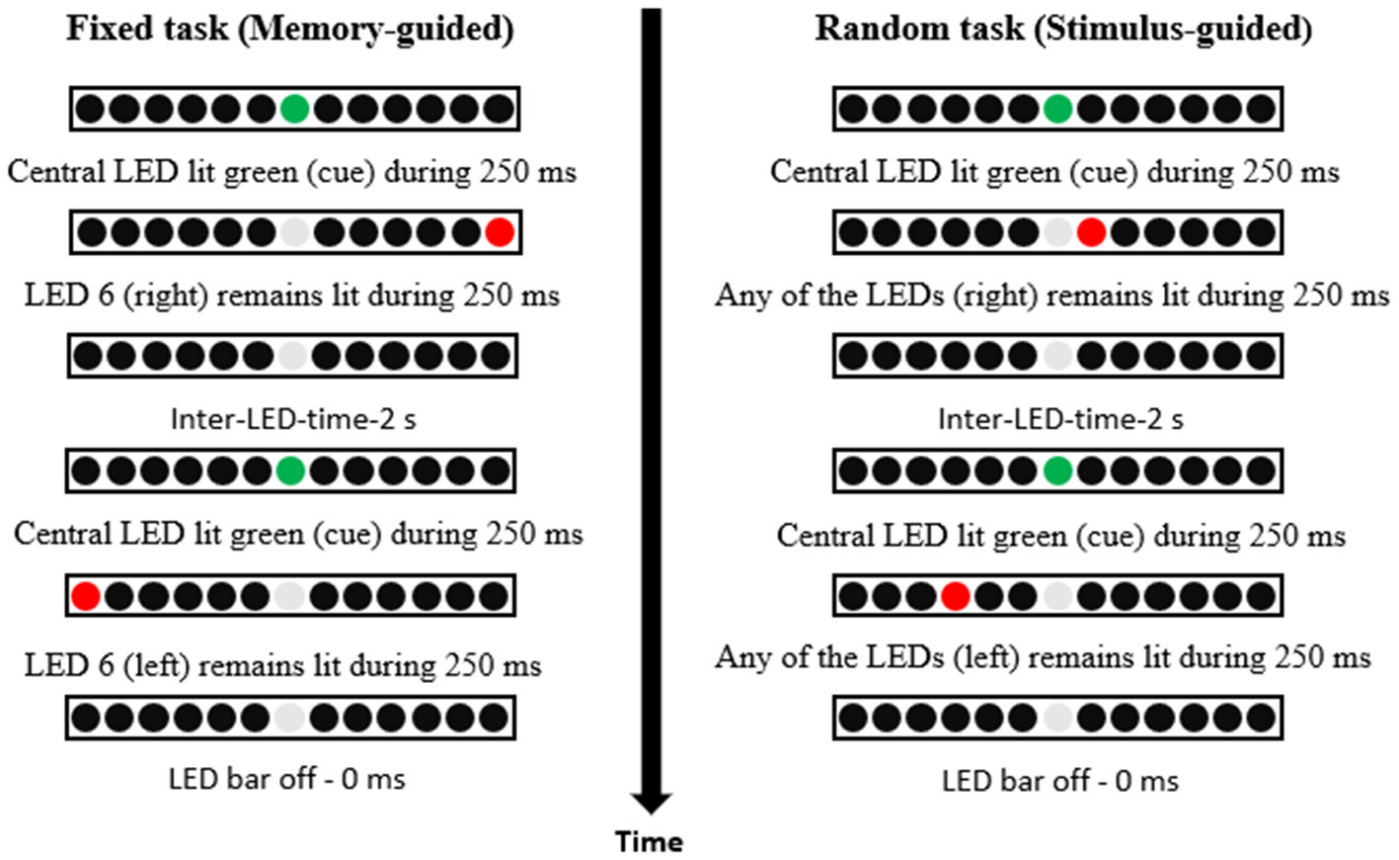
Illustration of the experimental time course of the two saccadic eye movement tasks. **(Left)** fixed task. **(Right)** random task.

Participants were instructed to fixate on the central LED and to shift their gaze to the target LED immediately upon its illumination, while avoiding head movements. The paradigm included two conditions: (i) a fixed task, in which the target LED (position 6) alternated predictably between left and right sides, always at 30° of visual angle; and (ii) a random task, in which any of the 12 peripheral LEDs could be illuminated in an unpredictable sequence, including central and peripheral positions. Each stimulus was presented for 250 ms with a 2 s interstimulus interval, see Figure 2. Participants completed 12 blocks (six per task) with 20 trials per block, and LED activation probability was counterbalanced within and across blocks.

### 2.3 Data acquisition

Continuous EEG was recorded using a 20-channel BrainNet BNT36 system (EMSA Medical Equipment) with silver/silver-chloride electrodes mounted on a nylon cap following the international 10–20 system and binaural reference electrodes (SPES Medical, Brazil). Participants sat in armrest chairs to minimize muscle artifacts. Electrode impedance was kept below 5 kΩ, and signal amplitude did not exceed 100 μV. Data were sampled at 240 Hz. Filters included a 100 Hz low-pass antialiasing filter, a 60 Hz digital notch filter, a 0.03 Hz high-pass filter, and a 40 Hz low-pass second-order Butterworth filter, implemented using Data Acquisition software (Delphi 5.0).

EEG signals were recorded as potential differences relative to the reference electrodes (earlobes) and were time-locked to stimulus presentation. For each participant, 15 epochs per condition were extracted from a 2 s window following stimulus onset (0 to 2 s post-stimulus).

### 2.4 EEG data processing

Artifact rejection was conducted by visual inspection and Independent Component Analysis (ICA) using EEGLAB (Delorme and Makeig, 2004) implemented in MATLAB. Electrodes showing poor contact or impedance above 5 kΩ were excluded prior to ICA decomposition. After ICA, less than 10% of the data were rejected per participant, regardless of task condition.

Power spectral density (PSD) was estimated using the Fourier Transform, computed via Bartlett's periodogram with non-overlapping 2 s rectangular windows (480 samples). After preprocessing, the total number of usable epochs was 376 for controls and 366 for volleyball athletes.

### 2.5 Statistical analysis

All analyses were performed in R (version 2025.05.1; R Core Team, 2025). The dependent variables were (i) saccadic latency and (ii) absolute alpha power. Saccadic onset was identified using a semi-automatic procedure based on the inflection point of the EOG curve and verified by visual inspection. The analysis window covered 500 ms after stimulus onset, and the point of maximum acceleration (second derivative of the EOG signal) was extracted. Saccades with latencies below 100 ms were classified as artifacts and excluded.

Assumption checks indicated violations of normality (Shapiro–Wilk, *p* < 0.05) and homogeneity of variances (Levene's test, *p* < 0.05); therefore, non-parametric analyses were employed. Data are presented as median and interquartile range (IQR), with whiskers extending to 3 × IQR. Main effects and interactions were assessed using the Scheirer–Ray–Hare test, a non-parametric analog of the two-way ANOVA (Scheirer et al., 1976). Effect sizes were calculated using epsilon-squared (ε^2^ = SS_effect/SS_total), the recommended effect size measure for rank-based non-parametric tests (Ialongo, 2016), and interpreted as small (ε^2^ = 0.01), medium (ε^2^ = 0.06), and large (ε^2^ = 0.14). When significant interactions occurred, Mann–Whitney tests were conducted, with Bonferroni correction applied for multiple electrode comparisons (adjusted α = 0.05/number of comparisons).

## 3 Results

This section details the findings for saccadic latency (behavioral measure) and absolute alpha power (electrophysiological parameter).

### 3.1 Saccadic latency

**Figure 3:**
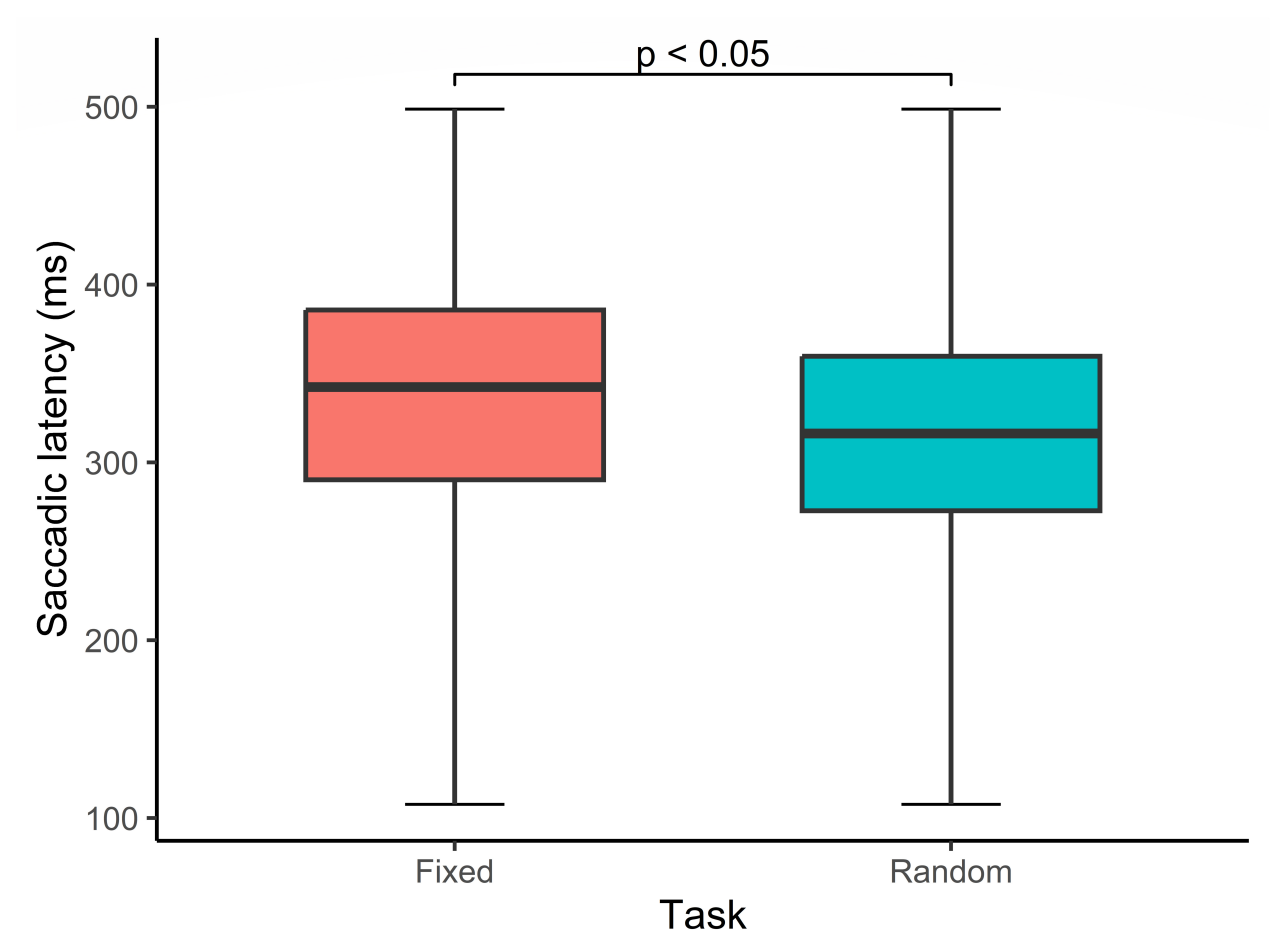
Saccadic latency in fixed and random tasks. Boxplots illustrate saccadic latency (ms) in fixed (memory-guided) and random (stimulus-guided) tasks. Data represents median, interquartile range (IQR), and minimum/maximum values, with whiskers extending up to 3.0 × IQR. Saccadic latency was significantly longer in the fixed task compared to the random task (*p* < 0.05).

The Scheirer–Ray–Hare test showed a significant main effect of Task [H(1) = 34.96, *p* < 0.001, ε^2^ = 0.023; Figure 3], with shorter saccadic latencies in the random task (median = 316 ms) than in the fixed task (median = 342 ms). No significant main effect of Group [H(1) = 2.83, *p* = 0.093, ε^2^ = 0.002] or Group × Task interaction [H(1) = 1.99, *p* = 0.159, ε^2^ = 0.001] was detected (Table 1).

**Table 1:** Results of the Scheirer–Ray–Hare test for saccadic latency.

Source	df	*H*	*p*
Group	1	2.83	0.093
Task	1	34.96	< 0.001
Interaction (Group × Task)	1	1.99	0.159

### 3.2 Absolute alpha power

**Figure 4:**
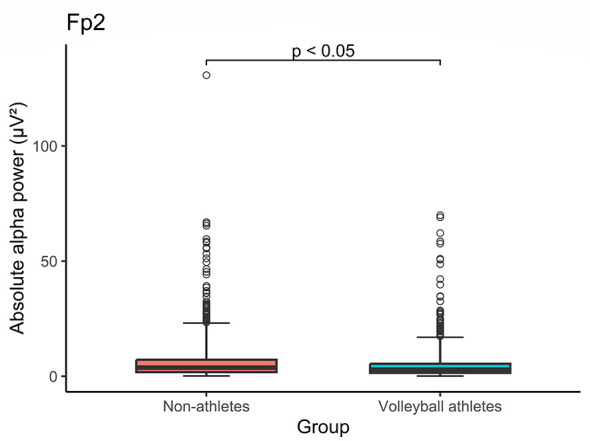
Absolute alpha power at Fp2 in non-athletes and volleyball athletes. Boxplots depicting absolute alpha power (μ*V*^2^) at Fp2 for non-athletes and volleyball athletes. Data represents median, interquartile range (IQR), and minimum/maximum values, with whiskers extending up to 3.0 × IQR. Significantly lower alpha power was observed in volleyball athletes compared to non-athletes (*p* < 0.05).

**Figure 5:**
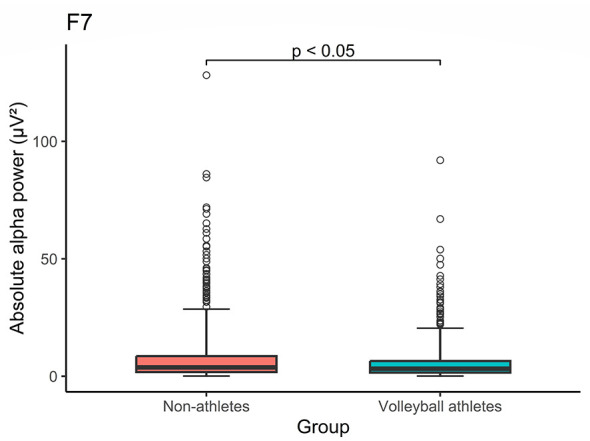
Absolute alpha power at F7 in non-athletes and volleyball athletes. Boxplots depicting absolute alpha power (μ*V*^2^) at F7 for non-athletes and volleyball athletes. Data represents median, interquartile range (IQR), and minimum/maximum values, with whiskers extending up to 3.0 × IQR. Significantly lower alpha power was observed in volleyball athletes compared to non-athletes (*p* < 0.05).

**Figure 6:**
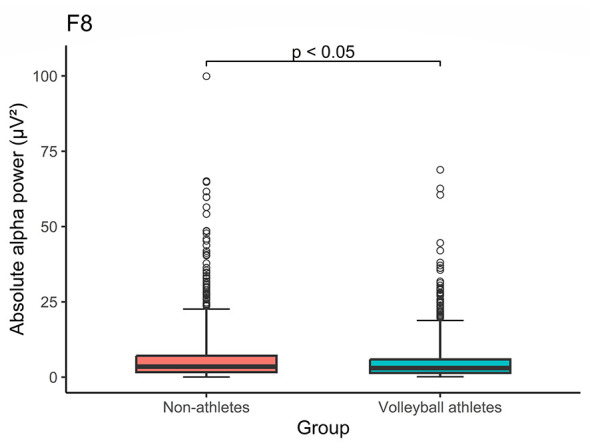
Absolute alpha power at F8 in non-athletes and volleyball athletes. Boxplots depicting absolute alpha power (μ*V*^2^) at F8 for non-athletes and volleyball athletes. Data represents median, interquartile range (IQR), and minimum/maximum values, with whiskers extending up to 3.0 × IQR. Significantly lower alpha power was observed in volleyball athletes compared to non-athletes (*p* < 0.05).

**Figure 7:**
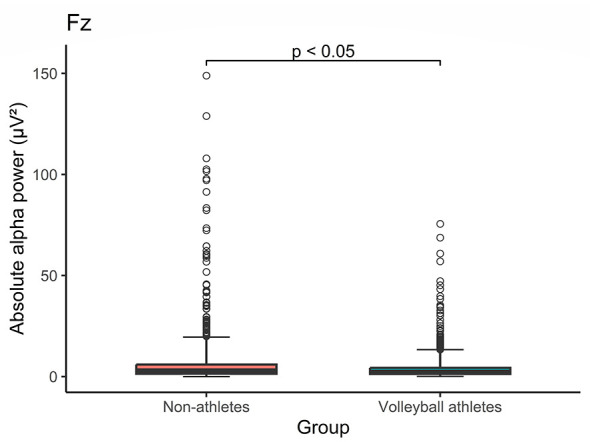
Absolute alpha power at Fz in non-athletes and volleyball athletes. Boxplots depicting absolute alpha power (μ*V*^2^) at Fz for non-athletes and volleyball athletes. Data represents median, interquartile range (IQR), and minimum/maximum values, with whiskers extending up to 3.0 × IQR. Significantly lower alpha power was observed in volleyball athletes compared to non-athletes (*p* < 0.05).

**Figure 8:**
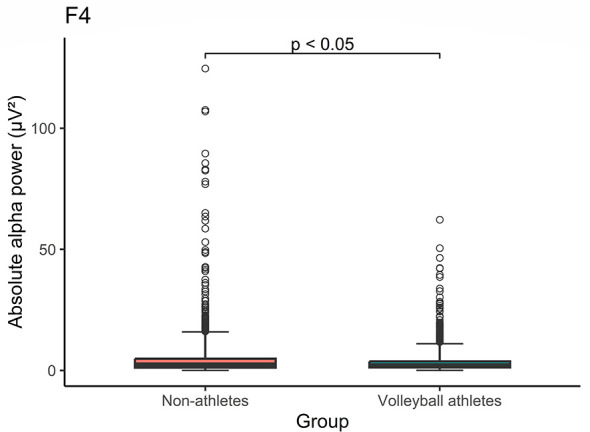
Absolute alpha power at F4 in non-athletes and volleyball athletes. Boxplots depicting absolute alpha power (μ*V*^2^) at F4 for non-athletes and volleyball athletes. Data represents median, interquartile range (IQR), and minimum/maximum values, with whiskers extending up to 3.0 × IQR. Significantly lower alpha power was observed in volleyball athletes compared to non-athletes (*p* < 0.05).

**Figure 9:**
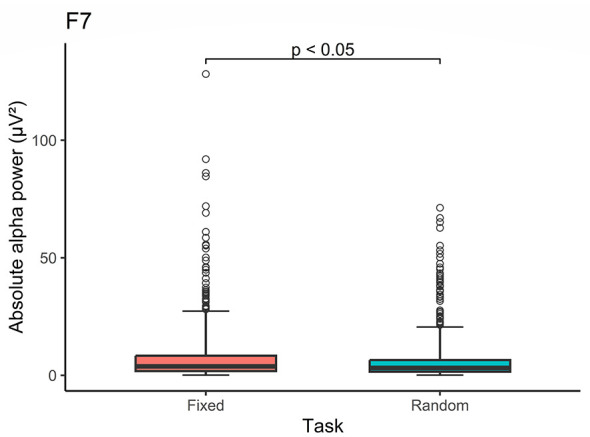
Absolute alpha power at F7 in fixed and random tasks. Boxplots illustrating absolute alpha power (μ*V*^2^) at F7 in fixed (memory-guided) and random (stimulus-guided) tasks. Data represents median, interquartile range (IQR), and minimum/maximum values, with whiskers extending up to 3.0 × IQR. Significantly lower alpha power was observed in the random task compared to the fixed task (*p* < 0.05).

**Figure 10:**
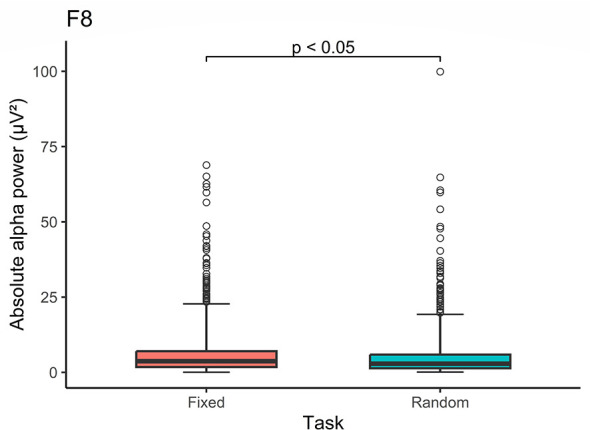
Absolute alpha power at F8 in fixed and random tasks. Boxplots illustrating absolute alpha power (μ*V*^2^) at F8 in fixed (memory-guided) and random (stimulus-guided) tasks. Data represents median, interquartile range (IQR), and minimum/maximum values, with whiskers extending up to 3.0 × IQR. Significantly lower alpha power was observed in the random task compared to the fixed task (*p* < 0.05).

Quantitative EEG analysis revealed a significant main effect of Group at Fp2 [H(1) = 46.14, p < 0.001, ε^2^ = 0.015; Figure 4], F7 [H(1) = 21.51, *p* < 0.001, ε^2^ = 0.007; Figure 5], F8 [H(1) = 19.19, *p* < 0.001, ε^2^ = 0.006; Figure 6], Fz [H(1) = 29.38, *p* < 0.001, ε^2^ = 0.009; Figure 7], and F4 [H(1) = 9.64, *p* = 0.002, ε^2^ = 0.003; Figure 8], with volleyball athletes showing lower median alpha power than non-athletes across these regions (Table 2). A significant main effect of Task was found at F7 [H(1) = 27.17, *p* < 0.001, ε^2^ = 0.009; Figure 9] and F8 [H(1) = 32.74, *p* < 0.001, ε^2^ = 0.011; Figure 10], with lower alpha power in the random task, reflecting greater neural engagement in the more complex condition. No significant Group × Task interactions were detected.

**Table 2:** Results of the Scheirer–Ray–Hare test for absolute alpha power.

Electrode/Source	df	*H*	*p*
Fp2—Group	1	46.14	< 0.001
Fp2—Task	1	20.59	< 0.001
Fp2—Interaction (Group × Task)	1	0.01	0.915
F7—Group	1	21.51	< 0.001
F7—Task	1	27.17	< 0.001
F7—Interaction (Group × Task)	1	0.02	0.896
F8—Group	1	19.19	< 0.001
F8—Task	1	32.74	< 0.001
F8—Interaction (Group × Task)	1	0.12	0.728
Fz—Group	1	29.38	< 0.001
Fz—Task	1	1.41	0.235
Fz—Interaction (Group × Task)	1	0.23	0.630
F4—Group	1	9.64	0.002
F4—Task	1	11.79	0.001
F4—Interaction (Group × Task)	1	1.66	0.198

## 4 Discussion

This study investigated saccadic latency and absolute alpha power during eye-movement preparation in volleyball athletes and non-athletes, using fixed (memory-guided) and random (stimulus-guided) tasks. Behavioral (latency) and electrophysiological (prefrontal and frontal alpha power) measures were analyzed with the Scheirer–Ray–Hare test to evaluate main and interaction effects.

### 4.1 Behavioral discussion

Statistical analysis revealed a significant main effect of Task, with shorter latencies in the random (316 ms) than in the fixed condition (342 ms). This pattern may be explained by two complementary mechanisms. First, unpredictable stimuli elicit greater phasic alertness, whereas the predictable and repetitive nature of the fixed task may induce monotony and reduce alertness, thereby increasing latencies. Second, in the random task, targets were presented across a range of eccentricities (5.5° to 30°), including positions closer to the central fixation point, while in the fixed task the target was always presented at 30°. Given that saccadic latency tends to decrease with target eccentricity, the broader range of eccentricities in the random condition may have contributed to the lower median latency observed. Contrary to previous reports of enhanced visual and perceptual-cognitive skills in athletes (Balser et al., 2014; Ghasemi et al., 2011; Meng et al., 2019; Piras et al., 2010), volleyball players did not show shorter saccadic latencies.

Elite athletes often show faster neural conduction in visual pathways, associated with greater white matter volume (Zwierko et al., 2010) and improved information processing (McDonough and Siegel, 2018). Although some studies report shorter saccadic latencies in athletes (Di Russo et al., 2003; Morrillo et al., 2006; Vicente et al., 2023; Yilmaz and Polat, 2018), others find no differences (Babu et al., 2005; Lenoir et al., 2000; van Maarseveen et al., 2018), suggesting that contextual and methodological factors influence results.

For example, studies using simple or non-sport-specific tasks often fail to reveal group differences (Del Percio et al., 2019; Nakazato et al., 2024), highlighting the importance of ecological validity and task complexity in detecting expertise effects. The exclusion of anticipatory saccades (latencies < 100 ms) may also have reduced between-group variability.

The fixed task, classified as memory-guided (Velasques et al., 2011), involves predictability and procedural learning, providing comparable familiarity for athletes and non-athletes (Bittencourt et al., 2012). This supports the idea that athletes' advantages in saccadic performance emerge mainly under unpredictable and cognitively demanding conditions (Bonney et al., 2025; Pinder et al., 2011).

Volleyball requires rapid responses to dynamic and unpredictable stimuli in complex environments (Paulo et al., 2016; Tomasino et al., 2012). The simplified experimental paradigm used here may have limited ecological validity, constraining the detection of expertise effects. The significant task effect, with shorter latencies in random conditions, suggests that unpredictability demands greater attentional engagement (DeCouto et al., 2023; Qiu et al., 2019).

This pattern suggests heightened sustained attention under unpredictable conditions, consistent with links between saccadic latency, early visual processing (Wang et al., 2025) and pre-motor attentional shifts (Topfstedt et al., 2023; Yu et al., 2024). This negative finding is informative and suggests that saccadic latency advantages in athletes emerge exclusively under ecologically-rich, game-realistic conditions, not simple lab paradigms. Future studies should employ more ecologically valid tasks to clarify the neural mechanisms underlying saccadic latency in athletes.

### 4.2 Electrophysiological discussion

This study investigated absolute alpha power differences between volleyball athletes and non-athletes across three prefrontal–frontal subregions: frontopolar (Fp1, Fp2), inferior prefrontal (F7, F8), and anterior frontal cortex (F3, Fz, F4). Analyses during the preparatory phase of fixed (memory-guided) and random (stimulus-guided) saccadic tasks revealed distinct neural activity patterns across groups and tasks, highlighting the neural correlates of expertise and visuomotor processing.

#### 4.2.1 Anterior prefrontal cortex—Fp1/Fp2

The Scheirer–Ray–Hare test revealed significant main effects of Group and Task at Fp1 and Fp2, with no Group × Task interaction. Volleyball athletes showed reduced absolute alpha power at Fp2, suggesting right-lateralized cortical engagement during visuomotor preparation. Activity recorded at Fp1/Fp2 may reflect, at least in part, contributions from frontopolar regions associated with high-level executive functions, including hierarchical action coordination, goal-directed motor planning, and attentional control (Braver and Bongiolatti, 2002; Chiang et al., 2013; Corbetta and Shulman, 2002; Domic-Siede et al., 2021); however, it is important to note that scalp EEG does not allow precise cortical source localization, and this interpretation should be considered with caution.

Reduced alpha power in athletes is seemingly at odds with the neural efficiency hypothesis, which predicts lower overall neural activation with expertise (Fang et al., 2022). However, lower alpha power reflects increased regional cortical activation rather than reduced effort (Poldrack, 2015), and recent evidence suggests that expertise involves selective regional specialization, with greater engagement of task-relevant networks and suppression of irrelevant ones, rather than global efficiency (Morrone and Pedlar, 2024; Shi et al., 2024). This is consistent with frontal alpha lateralization reported in elite athletes during specialized psychomotor tasks (Babiloni et al., 2009; Hatfield et al., 2004), and with evidence of heightened action-observation network activation in sport-specific contexts (Abreu et al., 2012; Kim et al., 2011; Wright et al., 2010).

The absence of a significant Task effect at Fp1/Fp2 suggests that prefrontal alpha activity was similarly engaged across fixed and random conditions, possibly reflecting stable executive monitoring demands regardless of stimulus predictability (Bittencourt et al., 2013; Morrone and Pedlar, 2024).

In summary, the right-lateralized reduction in alpha power at Fp2 in volleyball athletes indicates training-induced specialization of prefrontal attentional networks, a neural adaptation not captured by saccadic latency measures alone.

#### 4.2.2 Inferior prefrontal gyrus—F7/F8

The Scheirer–Ray–Hare test revealed significant main effects of both Group and Task at F7 and F8, with no Group × Task interaction. Volleyball athletes showed reduced absolute alpha power at these sites compared to non-athletes, and both groups exhibited lower alpha power during the random condition. Activity recorded at F7/F8 may reflect, at least in part, contributions from lateral prefrontal regions involved in attentional control, action observation, and visuomotor integration (Ardila et al., 2017; Binkofski and Buccino, 2006; Foundas et al., 1998); however, direct anatomical attribution is not possible given the spatial resolution of scalp EEG, and these associations should be interpreted accordingly.

Reduced alpha power at F7 and F8 in volleyball athletes is consistent with neural specialization observed in other sports (Song et al., 2024). Within the framework of alpha inhibition (Klimesch et al., 2007), lower alpha reflects the release of inhibitory constraints, facilitating focused attention on relevant visuomotor targets and supporting dynamic motor responses (Klimesch, 1999; Kuo et al., 2023). This pattern aligns with findings in visually demanding sports such as archery and shooting, where frontal alpha reductions during preparatory phases are consistently reported (Hatfield et al., 2004), and with neuroimaging evidence of greater lateral prefrontal activation in expert athletes compared to novices (Wu et al., 2013; Yu et al., 2019, 2023).

Regarding the Task effect, both groups showed lower alpha power at F7 and F8 during the random condition, suggesting that alpha oscillations are modulated by stimulus unpredictability regardless of athletic expertise (Corbetta and Shulman, 2002). This reduction likely reflects heightened visuomotor preparation and attentional allocation in response to unpredictable events (Kuo et al., 2023), consistent with the inhibition-timing hypothesis (Klimesch et al., 2007).

Notably, volleyball athletes maintained consistently lower alpha levels than non-athletes across both tasks, suggesting that training-induced neural adaptations persist independently of task demands. This persistent reduction may reflect the development of efficient attentional strategies for managing unpredictability, supporting rapid visual processing and decision-making under temporal pressure (Gu et al., 2022; Piras et al., 2010).

In summary, the group and task effects observed at F7 and F8 indicate that lateral prefrontal alpha activity is sensitive to both athletic expertise and stimulus predictability, reflecting training-induced specialization of attentional and visuomotor networks that is not apparent in behavioral measures alone.

#### 4.2.3 Anterior frontal cortex—F3/Fz/F4

The Scheirer–Ray–Hare test revealed significant main effects of Group at Fz and F4, and of Task at F4, with no Group × Task interaction at any of these sites. No significant effects were observed at F3. Activity recorded at F3, Fz, and F4 may reflect, at least in part, contributions from anterior frontal regions involved in saccadic planning, visuospatial attention, and executive control (Chuang et al., 2013; Teichert et al., 2014; Velasques et al., 2011); however, scalp EEG has limited spatial resolution, and the contribution of specific cortical regions cannot be determined with certainty.

The pattern of reduced alpha power at Fz and F4, but not F3, in volleyball athletes suggests possible right-hemisphere specialization in attentional and motor-preparatory processes. The right hemisphere is traditionally associated with visuospatial processing and global attentional control (Heilman and Van Den Abell, 1980), and volleyball requires simultaneous tracking of multiple dynamic targets, which places high demands on these functions (Mroczek et al., 2013; Paulo et al., 2016). Reduced alpha at F4 is consistent with training-induced right-lateralized adaptations reported in skilled volleyball players during attack anticipation (DeCouto et al., 2023), and with frontal alpha modulation linked to sensorimotor integration in other precision sports (Babiloni et al., 2008).

The absence of significant Task effects at F3, Fz, and F4 contrasts with the task sensitivity observed at F7 and F8, and parallels the pattern found at Fp1 and Fp2. This dissociation suggests that lateral frontal regions are more sensitive to stimulus predictability, whereas medial and central frontal regions primarily support general executive control, independent of task type (Kuo et al., 2023; Nakano et al., 2013; Sauseng et al., 2005). A similar functional dissociation has been reported in experienced golfers, where region-specific frontal activation during putting preparation reflects functional specialization within the frontal cortex (Milton et al., 2007).

In summary, the group effects observed at Fz and F4 indicate right-lateralized training-induced neural adaptations in volleyball athletes, reflecting enhanced visuospatial processing and attentional flexibility. The absence of task effects in these regions further supports the notion that medial and central frontal alpha activity reflects stable executive control demands, independent of stimulus predictability.

## 5 Conclusion

This study investigated differences between volleyball athletes and non-athletes in saccadic latency and absolute alpha power during eye-movement preparation using fixed (memory-guided) and random (stimulus-guided) tasks. The results offer complementary behavioral and electrophysiological insights into expertise-related visuomotor processing.

Behaviorally, saccadic latencies were shorter in the random than in the fixed task across groups, reflecting heightened attentional demands under unpredictable conditions. No significant group differences were observed, likely due to the limited sensitivity of the fixed task for capturing expertise effects. Electrophysiologically, volleyball athletes consistently exhibited lower frontal and prefrontal alpha power, indicating distinct neural activation patterns relative to non-athletes.

These results indicate that sports expertise can emerge at subtle neural levels undetectable by conventional reaction-time measures, underscoring EEG as a sensitive tool for identifying training-induced neural adaptations. Collectively, the findings advance understanding of the neurophysiological mechanisms underlying visuomotor performance and highlight the modulatory influence of sports training on brain activity.

Despite these findings, several limitations should be considered. The simplicity of the tasks may have restricted the detection of behavioral differences related to sports expertise, as evidenced by the lack of group effects on saccadic latency. This likely reflects the low ecological validity of the paradigm, which did not fully engage the perceptual–motor skills required in real-game situations. Additionally, excluding saccadic latencies below 100 ms to remove anticipatory responses may have influenced the results. Future research should use more ecologically valid tasks that better represent actual gameplay dynamics.

A formal power analysis was not conducted prior to data collection, which represents a limitation of the present study. The sample size was based on previous studies with similar designs (Del Percio et al., 2007b; Di Russo et al., 2005b). A larger sample (*N* ≥ 60) would provide greater statistical power to detect smaller between-group differences, and future longitudinal studies with larger cohorts are recommended to validate these preliminary neuroplasticity markers.

Another limitation concerns the spatial resolution of EEG. While the 10–20 system is widely used, it restricts precise cortical source localization. Future research should employ higher-density electrode arrays (e.g., 10–10 or 10–5 systems) and source reconstruction methods to enhance spatial interpretation of neural activity.

In summary, despite these limitations, the findings are robust and point to promising directions for methodological refinement in sports neuroscience. Longitudinal studies and cross-sport comparisons provide valuable opportunities to map training-related neural adaptations and advance the field.

## Data Availability

The data that support the findings of this study are available from the corresponding author upon reasonable request.
